# Neoadjuvant Chemotherapy *Versus* Direct Surgery for Locally Advanced Gastric Cancer With Serosal Invasion (cT4NxM0): A Propensity Score-Matched Analysis

**DOI:** 10.3389/fonc.2021.718556

**Published:** 2021-08-23

**Authors:** Wei Xu, Lingquan Wang, Chao Yan, Changyu He, Sheng Lu, Zhentian Ni, Zichen Hua, Zhenglun Zhu, Birendra Kumar Sah, Zhongyin Yang, Yanan Zheng, Runhua Feng, Chen Li, Xuexin Yao, Mingmin Chen, Wentao Liu, Min Yan, Zhenggang Zhu

**Affiliations:** Department of General Surgery, Shanghai Key Laboratory of Gastric Neoplasms, Shanghai Institute of Digestive Surgery, Ruijin Hospital, Shanghai Jiao Tong University School of Medicine, Shanghai, China

**Keywords:** neoadjuvant chemotherapy, perioperative chemotherapy, locally advanced gastric cancer, gastrectomy, propensity score matching, real-world study

## Abstract

**Background:**

For locally advanced gastric cancer (LAGC) with serosal invasion (cT4NxM0), adjuvant chemotherapy (AC) after D2 gastrectomy is the standard therapy in Asia. However, perioperative chemotherapy (PCT) combined with D2 gastrectomy is mostly suggested in Europe and America. As a part of PCT, the value of neoadjuvant chemotherapy (NAC) is unclear. We investigated whether NAC could further improve survival and other outcomes for these patients.

**Methods:**

Patients with cT4NxM0 gastric cancer who underwent D2 gastrectomy were analyzed. The patients were divided into two groups based on whether they received NAC: the neoadjuvant chemotherapy (NAC) and direct surgery (S) groups. After propensity score matching (1:1 ratio), survival and perioperative outcomes were analyzed between the two groups.

**Results:**

A total of 902 patients met all the eligibility criteria and were enrolled. After propensity score matching, 221 matched pairs of patients were identified. The median overall survival (OS) and disease-free survival (DFS) of all patients were 75.10 and 43.67 months, respectively. The median OS of patients in the NAC and S groups were undefined and 29.80 months, respectively (P<0.0001). The median DFS of patients in the NAC and S groups were undefined and 22.60 months (P<0.0001). There were no significant differences in the radical degrees of operation between the two groups (P=0.07). However, there were significant differences in postoperative hospital stay (P<0.001) and complications (P=0.037) between the two groups.

**Conclusion:**

This study suggested NAC can further improve prognosis and prevent recurrence in LAGC (cT4NxM0) patients. NAC is feasible and safe for LAGC (cT4NxM0) patients, and does not increase the risk of perioperative surgery.

## Introduction

Gastric cancer (GC) is one of the most common malignant tumors worldwide, with a high incidence and mortality rate. GC is the fifth most common cancer and the third leading cause of cancer-related deaths worldwide (1). In China, GC is the second most common cancer and the second leading cause of cancer death (2). Different stages of GC have different biological behaviors, treatment strategies and prognoses. For early gastric cancer (EGC), the primary treatment option is surgery (3–7). For advanced gastric cancer (AGC) with distant metastasis, comprehensive treatment based on systemic antitumor therapy is recommended to prolong the survival and improve the quality of life of patients (8–10). For locally advanced gastric cancer (LAGC), over the past few decades, the standard therapy has been D2 gastrectomy followed by adjuvant chemotherapy (AC), which was confirmed by several randomized controlled trials (RCTs) to improve disease-free survival (DFS) and overall survival (OS) compared with surgery alone (11, 12). The ACTS-GC and CLASSIC studies showed that AC with S-1 or XELOX could improve OS and DFS in patients with LAGC who had undergone curative D2 gastrectomy (11–14).

In recent years, more attention has been given to perioperative chemotherapy (PCT). PCT was widely accepted until a series of RCTs were performed to evaluate its value (15–17). The MAGIC trial was the first to show a survival benefit of surgery combined with PCT. The MAGIC trial showed that PCT with the ECF regimen decreased tumor size and stage and improved PFS and OS in patients with LAGC (15). However, less than 50% patients in the MAGIC trial underwent a D2 resection. Another RCT study (FNCLCC & FFCD trial) showed that PCT could increase the curative resection rate, DFS and OS in patients with LAGC (16). The two trials showed that PCT, on the basis of surgery, could further increase the 5-year OS rate by approximately 13~14% in LAGC. However, as a part of PCT, the value of neoadjuvant chemotherapy (NAC) in improving OS and DFS is unclear. It is unknown whether PCT is better than AC for LAGC patients who undergo D2 gastrectomy.

NAC is performed preoperatively and could result in disease progression during treatment. Although NAC has some theoretical advantages (15), it is unknown whether NAC could further improve the survival of LAGC on the basis of D2 gastrectomy followed by AC. Therefore, there are two ongoing RCTs addressing this issue, which were reported at the European Society for Medical Oncology (ESMO) 2019 conference. The PRODIGY study showed that the 3-year and 5-year DFS rates in the NAC (NAC + surgery + AC) group were significantly higher than those in the S (surgery +AC) group (18). The RESOLVE study showed that PCT improved the 3-year DFS rate compared with AC alone (19). In summary, PCT, the combination of AC and NAC, could increase the 3-year DFS rate by approximate 6~7% in LAGC compared with AC alone.

However, the value of NAC itself for LAGC patients in improving OS has not been reported. In China, NAC has not been used for all LAGC patients. Currently, NAC is mainly used in LAGC with serosal invasion (cT4NxM0). Therefore, we conducted our study to investigate whether the addition of NAC can further improve OS and other outcomes of LAGC (cT4NxM0) patients.

## Materials and Methods

### Patient Selection

From our electronic medical record system which included all patients admitted to our gastric cancer professional group, we investigated 3228 patients with primary gastric cancer and without a history of other malignancies at Ruijin Hospital (Shanghai Jiao Tong University School of Medicine, Shanghai, China) between January 2013 and December 2018. The inclusion criteria were as follows: (1) pathologically proven gastric adenocarcinoma by gastroscopy before any treatment, (2) patients aged under 80 years old at their first gastroscopy, (3) patients without any antitumor therapy, (4) patients who provided consent for our treatment, (5) patients with pretreatment CT in our hospital, (6) patients with serosal invasion and without distant metastasis (cT4NxM0), (7) patients with no digestive tract obstruction, (8) patients with no active gastrointestinal bleeding, and (9) patients who underwent D2 gastrectomy and AC. Patients with clinical T stage 1~3, distant metastases, or changes in therapy regimen or without gastrectomy and AC were excluded from our study. According to whether the patients received NAC, all enrolled patients were divided into two groups: the NAC (NAC + surgery + AC) and S (surgery + AC) groups. The main difference between the two groups was the presence or absence of NAC.

In our database, we collected some pre-treatment information of patients, including sex, age, body mass index (BMI), hemoglobin, platelet, leukocyte, pre-albumin, total protein, albumin, blood tumor indicators (CA125, CA199, CA724, CEA, AFP), tumor differentiation, signet ring cell carcinoma component, Borrmann type and clinical TNM stage. Considering that there may be differences in baseline characteristics between the NAC and S groups, we performed propensity score matching analysis to match the NAC group to the S group at a ratio of 1:1.

Besides, we also collected some information during and after treatment, including therapy regimen, radical degrees of operation, postoperative complications, postoperative hospital stay, pathological TNM stage, disease recurrence time and death time. The radical degrees of operation were classified into three degrees: R0, macroscopically complete surgical resection with negative microscopic margins; R1, macroscopically complete surgical resection with positive microscopic margins; R2; macroscopically incomplete surgical resection.

This study was performed with the approval of the Ethics Committee of Ruijin Hospital affiliated to Shanghai Jiao Tong University School of Medicine. All patients were enrolled after signing an informed consent form.

### Neoadjuvant Chemotherapy

In the NAC group, patients received NAC before D2 gastrectomy followed by AC. We performed NAC based on the guidelines of the National Comprehensive Cancer Network (NCCN) and Chinese Society of Clinical Oncology (CSCO). Due to the progress of new RCT research, the guidelines and NAC regimens have also changed over time. Even so, NAC regimens are still based on the combination of 5-fluorouracil (5-FU) and platinum drugs, such as EOX (Epirubicin, Oxaliplatin and Capecitabine), XELOX (Oxaliplatin and Capecitabine), SOX (Oxaliplatin and S-1) and FLOT (Docetaxel, Oxaliplatin, Fluorouracil, and Leucovorin). All patients in the NAC group received average 3~4 cycles NAC. Before each cycle of NAC, patients were tested for hematological indicators, including blood routine, liver function, renal function, electrolyte, DIC and tumor markers.

### Evaluation of Neoadjuvant Chemotherapy

There are two methods to evaluate the response to NAC in LAGC: imaging and pathology. Before surgery, the response to NAC can be assessed by imaging evaluation criteria. The most commonly used imaging evaluation criteria is the Response Evaluation Criteria In Solid Tumor (RECIST 1.1) (20), in which the response to NAC is divided into four grades: complete response (CR), partial response (PR), stable disease (SD), and progressive disease (PD).

After surgery, we assessed the response to NAC in the NAC group through pathological evaluation criterion. The tumor regression grade (TRG) system is an effective pathology evaluation criterion. There are several TRG systems used to assess the tumor pathological response to NAC, including the Mandard, Ninomiya, Becker and Ryan classification systems (21–24). In our study, we used the Ryan classification system, which is the most widely applied by the College of American Pathologists (CAP) and the Chinese Society of Clinical Oncology (CSCO), to assess the pathological response of tumors to NAC (8, 25). The TRG classification system is divided into four categories: grade 0 (complete response: no viable cancer cells), grade 1 (moderate response: single cells or small groups of cancer cells), grade 2 (minimal response: residual cancer outgrown by fibrosis) and grade 3 (poor response: minimal or no tumor cells killed; extensive residual cancer).

### Surgery

For all enrolled patients in both the NAC and S groups, we performed D2 gastrectomy. All surgery were performed by the same surgical team of the gastric cancer specialized group in Ruijin hospital. The range of gastric resection and the method of reconstruction were determined by the patient’s tumor location. Distal gastrectomy was the first choice for distal gastric cancer, and Billroth I stomach-duodenal anastomosis, Billroth II stomach-jejunal anastomosis or Roux-en-Y stomach-jejunal anastomosis could be used for reconstruction. Total gastrectomy was the first choice for proximal gastric cancer, and Roux-en-Y esophagus-jejunal anastomosis was used for reconstruction. No prophylactic splenectomy is performed in either distal gastrectomy or total gastrectomy. If the primary tumor involves spleen, transverse colon, pancreas, left liver and other organs around the stomach, combined organ resection should be decided by the same surgical team. Postoperative complications were graded using the Clavien-Dindo Complications Classification (CDCC) (26). In this study, postoperative complications of grade III or above were recorded.

### Adjuvant Chemotherapy and Follow-Up

All patients received postoperative adjuvant chemotherapy. All chemotherapy regimens were based on NCCN and CSCO guidelines. There was no significant difference in chemotherapy regimens between the two NAC and S groups. The regimens of AC were basically based on 5-FU and platinum drugs.

### Follow-Up

We followed up with the patients through outpatient visits and telephone calls. Outpatient follow-up mainly included physical examination, hematological examination, multidetector computed tomography (MDCT) and gastroscopy. Magnetic resonance imaging (MRI) and positron emission tomography/computed tomography (PET/CT) scan were additionally performed when necessary. Telephone follow-up was conducted almost every three months within two years after surgery. After two years, telephone follow-up was conducted every 6 months. The date of death and the first relapse were recorded. The primary endpoint of this study was the overall survival (OS). Disease-free survival (DFS) was the secondary endpoint. OS was measured from the date of initial diagnosis of gastric cancer to the date of death or the last follow-up. DFS was defined as the time from the date of D2 gastrectomy to the recurrence of gastric cancer or the last follow-up.

### Statistical Analysis

To analyze the significance of enumeration data, chi-square test was used. For the measurement data, t-tests or the Mann-Whitney rank tests were used. Based on the differences between the NAC and S groups, we performed propensity score matching analysis to match the NAC group to the S group at a ratio of 1:1. We performed an exact match for region and used 2% caliper matching for the propensity score for the other variables. The Kaplan-Meier method was used to generate survival curves and analyze OS and DFS. All statistical tests were two-tailed, and the differences were statistically significant at P<0.05. Analyses were performed with SPSS version 26.0 (IBM Statistical Product and Service Solutions, Armonk, USA). GraphPad Prism version 8.0 (GraphPad, San Diego, CA, USA) was used to draw the survival curve and to calculate the survival rate and the median survival time.

## Results

### Characteristics of the Patients

**Figure 1:**
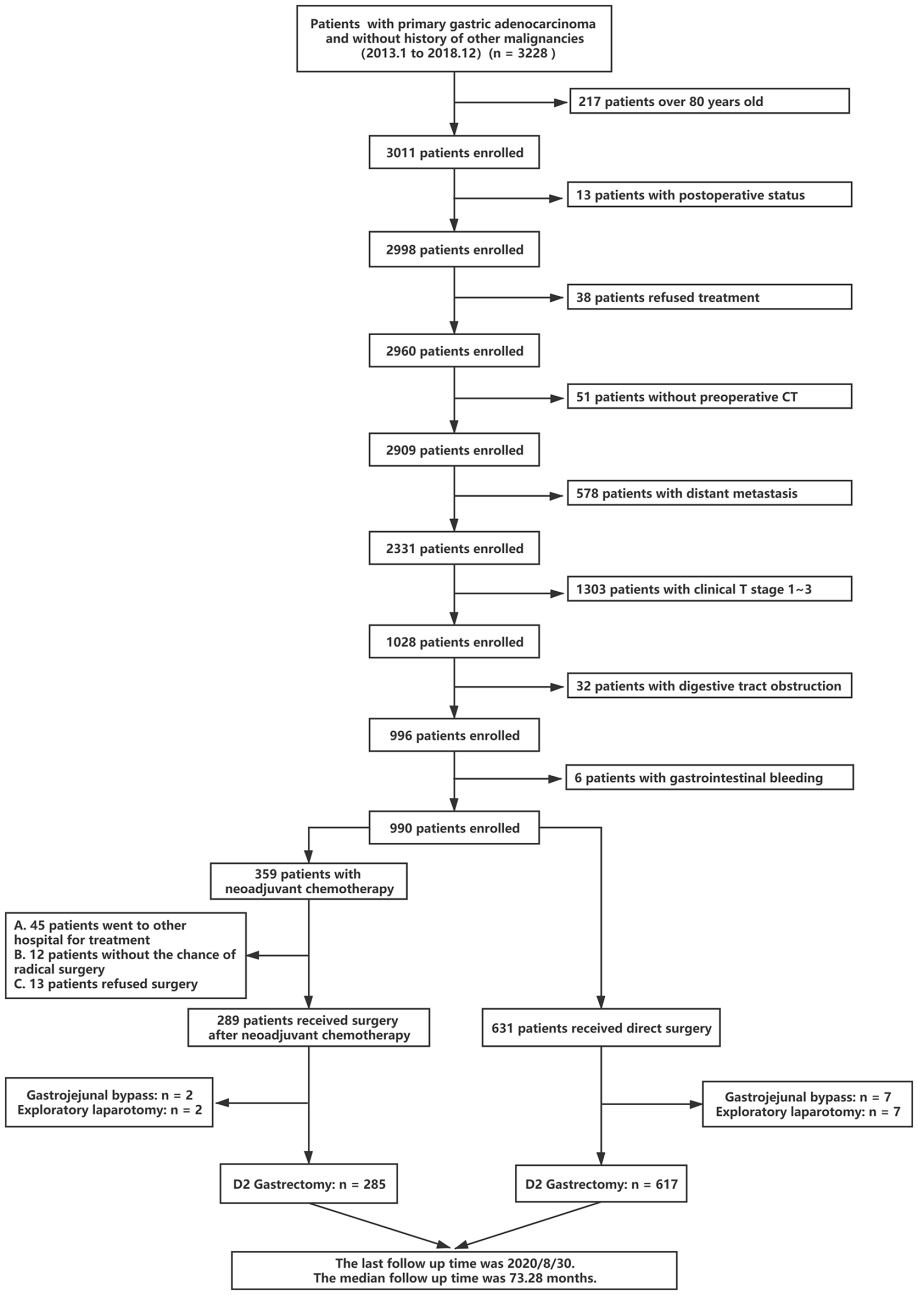
Flowchart of patient selection process.

From January 2013 to December 2018, a total of 902 patients satisfied all the eligibility criteria and 2326 patients were excluded from the study (**Figure 1**). The last follow-up date was 30 August 2020, and the median follow-up time was 73.28 months (range 0.40 - 93.50 months). Of the 902 patients, 375 patients (41.57%) had died of GC, and 455 patients had experienced recurrence (50.44%) by the last follow-up day. A total of 51 (5.65%) patients were lost during the follow-up period. Of all eligible patients, 285 patients (31.60%) received NAC, and 617 patients (68.40%) underwent D2 gastrectomy followed by AC alone. The pretreatment clinical characteristics of the 902 patients are summarized in **Table 1**. Between the NAC and S groups, several baseline characteristics had significant differences (P<0.05), including platelet, albumin, CA125, CA724, CEA, tumor differentiation, signet ring cell carcinoma component, Borrmann type and clinical N stage (**Table 1**). Three tumor markers (CA125, CA724 and CEA) in the NAC group were significantly higher than those in the S group (P<0.01). In the NAC group, there were 269 (94.39%) patients with Borrmann III/IV, which was significantly more than that in the S group (544, 88.17%, P<0.01). Regarding clinical N stage, in the NAC group, there were 61 (21.40%) patients with N0-1 stage disease, 139 (48.77%) patients with N2 stage disease and 85 (29.82%) patients with N3 stage disease. In the S group, there were 343 (55.59%) patients with N0-1 stage, 227 (36.79%) patients with N2 stage and 47 (7.62%) patients with N3 stage. These significant differences showed that patients in the NAC group experienced a heavier tumor burden and advanced disease, which were associated with poor prognosis and could affect the OS and DFS of patients (27, 28). On the other hand, there were more well-differentiated tumors in the NAC group than the S group (44.21% *vs* 33.39%, P<0.01). In addition, there were fewer patients with signet ring cell carcinoma components in the NAC group than in the S group (21.05% *vs* 39.55%, P<0.001). It seemed that patients in the NAC group had better tumor differentiation which was considered to be associated with a better response to chemotherapy (29).

**Table 1:** Pretreatment clinical characteristics of LAGC (cT4NxM0) patients before 1:1 matched.

Characteristics	Total (N = 902)	NAC (n = 285)	S (n = 617)	P
**Sex (n[%])**				0.050*
Male	618 (68.51)	208 (72.98)	410 (66.45)	
female	284 (31.49)	77 (27.02)	207 (33.55)	
**Age (y)**				0.624^§^
Median (range)	62 (21-80)	63 (21-80)	62 (26-80)	
**BMI (kg/m^2^)**				0.358^#^
Median (range)	22.80 (13.97-33.20)	22.91 (14-33.20)	22.72 (13.97-32.89)	
**Hemoglobin (g/L)**				0.446^§^
Median (range)	124 (44-184)	123 (44-164)	124 (45-184)	
**Leukocyte (10^9/L)**				0.200^§^
Median(range)	5.70 (2.20-16.90)	5.70 (2.40-16.90)	5.70 (2.20-14.93)	
**Platelet (10^9/L)**				0.034^§^
Median (range)	216 (41-924)	223 (82-924)	211 (41-754)	
**Prealbumin (g/L)**				0.192*
Median (range)	208 (67-388)	206 (79-354)	211 (67-388)	
**Total Protein (g/L)**				0.990^§^
Median (range)	65 (41-82)	64 (46-78)	65 (41-82)	
**Albumin (g/L)**				0.040^§^
Median (range)	37 (21-48)	37 (21-47)	37 (21-48)	
**CA125 (U/mL)**				0.001^§^
Median (range)	10.90 (1.20-601.80)	12.10 (2.19-314.10)	10.25 (1.20-601.80)	
**CA199 (U/mL)**				0.646^§^
Median (range)	8.20 (0.80-20830)	8 (0.80-7424)	8.20 (0.80-20830)	
**CA724 (U/mL)**				0.005^§^
Median (range)	2.33 (0.06-300)	3.19 (0.06-300)	2.14 (0.20-300)	
**CEA (ng/mL)**				0.002^§^
Median (range)	2.24 (0.50-1803.83)	2.42 (0.50-1400.45)	2.16 (0.50-1803.83)	
**AFP (ng/mL)**				0.760^§^
Median (range)	2.55 (0.50-10783.52)	2.54 (0.65-10783.52)	2.56 (0.50-9017.75)	
**Differentiation (n[%])**				0.002*
Well	332 (36.81)	126 (44.21)	206 (33.39)	
poor	570 (63.19)	159 (55.79)	411 (66.61)	
**Signet ring cell (n[%])**				<0.001*
Yes	304 (33.70)	60 (21.05)	244 (39.55)	
No	598 (66.30)	225 (78.95)	373 (60.45)	
**Borrmann (n[%])**				0.004*
I/II	89 (9.87)	16 (5.61)	73 (11.83)	
III/IV	813 (90.13)	269 (94.39)	544 (88.17)	
**cN stage (n[%])**				<0.001*
0-1	404 (44.79)	61 (21.40)	343 (55.59)	
2	366 (40.58)	139 (48.77)	227 (36.79)	
3	132 (14.63)	85 (29.82)	47 (7.62)	

*χ^2^ test (compares the counts of categorical responses between 2 or more independent groups).

^§^Mann-Whitney rank test (a nonparametric alternative to the 2 sample t test compares the means of 2 independent groups).

^#^T test (compare the means of 2 independent groups).

### Propensity Score Matching Analysis

Owing to the differences in baseline characteristics between the NAC and S groups, we performed propensity score matching analysis to match the NAC group to the S group at a ratio of 1:1. There were 18 baseline parameters used for propensity score matching, including sex, age, BMI, hemoglobin, platelet, leukocyte, pre-albumin, total protein, albumin, CA125, CA199, CA724, CEA, AFP, tumor differentiation, signet ring cell carcinoma component, Borrmann type and clinical N stage. After propensity score match analysis, 221 matched pairs of patients were identified. There were no significant differences between the two groups. The comparison of the two groups is shown in **Table 2**. Between the 221 matched pairs of patients, there were 331 males and 111 females, with a male-to-female ratio of 2.98:1. The median age at diagnosis was 62.50 (range: 21-80) years. In the NAC group, 148 (66.97%) and 73 (33.03%) patients achieved PR and SD, respectively. In addition, twenty patients obtained TRG 0 grade. For the regimens of NAC, 142 patients received EOX, 59 received SOX, 4 received XELOX, and 16 received FLOT.

**Table 2:** Pre-treatment clinical characteristics of LAGC (cT4NxM0) patients after 1:1 matched.

Characteristics	Total (N = 442)	NAC (n = 221)	S (n = 221)	P
**Sex (n[%])**				0.443*
Male	331 (74.89)	162 (73.30)	169 (76.47)	
female	111 (25.11)	59 (26.70)	52 (23.53)	
**Age (y)**				0.350^§^
Median (range)	62.50 (21-80)	63 (21-80)	61 (36-80)	
**BMI (kg/m^2^)**				0.741^#^
Median (range)	22.90 (14-33.20)	22.84 (14-33.20)	22.99 (14.98-31.59)	
**Hemoglobin (g/L)**				0.413^§^
Median (range)	123 (44-173)	125 (44-164)	121 (45-173)	
**Leukocyte (10^9/L)**				0.251^§^
Median(range)	5.80 (2.30-16.90)	5.70 (2.70-16.90)	5.80 (2.30-14.93)	
**Platelet (10^9/L)**				0.311^§^
Median (range)	218.50 (56-875)	216 (88-875)	226 (56-754)	
**Prealbumin (g/L)**				0.247^#^
Median (range)	206.50 (92-366)	203 (118-340)	212 (92-366)	
**Total Protein (g/L)**				0.953^§^
Median (range)	64 (41-82)	64 (46-77)	64 (41-82)	
**Albumin (g/L)**				0.712^§^
Median (range)	37 (21-48)	37 (21-47)	37 (21-48)	
**CA125 (U/mL)**				0.111^§^
Median (range)	10.95 (2.19-465.20)	11.60 (2.19-314.10)	10.40 (2.90-465.20)	
**CA199 (U/mL)**				0.488^§^
Median (range)	9.45 (0.80-7424)	8.70 (0.80-7424)	10.1 (0.80-3842.20)	
**CA724 (U/mL)**				0.419§
Median (range)	2.83 (0.46-300)	3.36 (0.46-300)	2.43 (0.66-300)	
**CEA (ng/mL)**				0.356^§^
Median (range)	2.40 (0.50-1400.45)	2.42 (0.50-1400.45)	2.38 (0.50-930.43)	
**AFP (ng/mL)**				0.326^§^
Median (range)	2.60 (0.77-9017.75)	2.46 (0.90-3220.19)	2.66 (0.77-9017.75)	
**Differentiation (n[%])**				0.702*
Well	196 (44.34)	100 (45.25)	96 (43.44)	
poor	246 (55.66)	121 (54.75)	125 (56.56)	
**Signet ring cell (n[%])**				0.586*
Yes	113 (25.57)	54 (24.43)	59 (26.70)	
No	329 (74.43)	167 (75.57)	162 (73.30)	
**Borrmann (n[%])**				0.208*
I/II	24 (5.43)	15 (6.79)	9 (4.07)	
III/IV	418 (94.57)	206 (93.21)	212 (95.93)	
**cN stage (n[%])**				0.742*
0-1	118 (26.70)	57 (25.79)	61 (27.60)	
2	244 (55.20)	126 (57.01)	118 (53.39)	
3	80 (18.10)	38 (17.19)	42 (19.01)	

*χ^2^ test (compares the counts of categorical responses between 2 or more independent groups).

^§^Mann-Whitney rank test (a nonparametric alternative to the 2 sample t test compares the means of 2 independent groups).

^#^T test (compare the means of 2 independent groups).

### Survival Analysis

**Figure 2:**
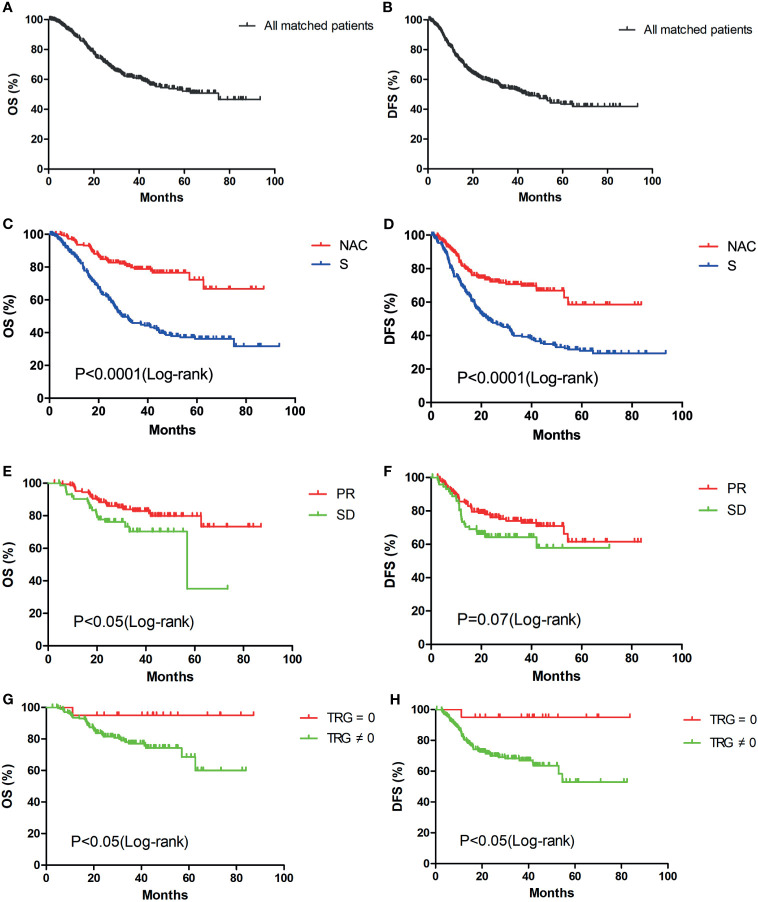
Kaplan-Meier survival curves of overall survival and disease-free survival: OS **(A)** and DFS **(B)**analysis of all matched patients (n=442); OS **(C)** and DFS **(D)** analysis of patients in the NAC (n=221) and S (n=221) groups; OS **(E)** and DFS **(F)** analysis of patients in the PR (n=148) and SD (n=73) groups; OS **(G)** and DFS **(H)** analysis of patients in the TRG = 0 (n=20) and TRG ≠0 (n=201) groups.

Among the 442 matched patients, after a median follow-up of 53.25 months, 172 patients (38.91%) had died of gastric cancer and 206 patients had experienced disease recurrence (46.61%) by the last follow-up day. There were 66 and 140 patients with disease recurrence in the NAC and S groups, respectively. The details of the recurrence sites which were first found had been shown in **Table 3**. A total of 26 (5.88%) patients were lost during the follow-up period. The median overall survival of the patient population was 75.10 months (**Figure 2A**), and the median disease-free survival was 43.67 months (**Figure 2B**). The median OS of patients in the NAC and S groups was undefined and 29.80 months, respectively (P<0.0001, HR 0.34, 95% CI 0.25–0.46, **Figure 2C**). The 1-year, 3-year, and 5-year OS rates for patients in the NAC group were 93.59%, 78.82% and 72.29%, respectively. For patients in the S group, the 1-year, 3-year, and 5-year OS rates were 83.71%, 45.90% and 36.22%, respectively. In addition, the median DFS of patients in the NAC and S groups was undefined and 22.60 months, respectively (P<0.0001, HR 0.44, 95% CI 0.33 - 0.58, **Figure 2D**). The 1-year, 3-year, and 5-year DFS rates for patients in the NAC group were 82.53%, 69.74% and 58.53%, respectively. For patients in the S group, the 1-year, 3-year, and 5-year DFS rates were 70.44%, 39.86% and 30.87%, respectively.

**Table 3:** Details of first recurrence site.

First Recurrence Site	Total (N = 206)	NAC (n = 66)	S (n = 140)
**Local recurrence (n[%])**	10 (4.85)	5 (7.58)	5 (3.57)
**Distant recurrence (n[%])**			
Peritoneal	149 (72.33)	42 (63.64)	107 (76.43)
Liver	17 (8.25)	7 (10.61)	10 (7.14)
Systemic lymph node	9 (4.37)	4 (6.06)	5 (3.57)
Ovarian	4 (1.94)	1 (1.52)	3 (2.14)
Bone	2 (0.97)	0 (0.00)	2 (1.43)
Multiple organs	15 (7.28)	7 (10.61)	8 (5.71)

Of all 902 patients, no patient achieved CR, and only 1 patient achieved PD after NAC. During propensity score matching, the PD patient in the NAC group was not matched in the S group. Therefore, according to the RECIST standard, 148 (66.97%) and 73 (33.03%) patients in the NAC group achieved PR and SD, respectively. We compared the survival between the PR and SD groups. The median OS of patients in the PR and SD groups was undefined and 56.97 months, respectively (P<0.05, **Figure 2E**). The 1-year, 3-year, and 5-year OS rates for patients in the PR group were 95.21%, 82.81% and 79.82%, respectively. For patients in the SD group, the 1-year, 3-year, and 5-year OS rates were 90.28%, 70.19% and 35.09%, respectively. The median DFS for PR and SD patients was undefined and was not significantly different (P=0.07, **Figure 2F**). The 1-year, 3-year, and 5-year DFS rates for patients in the PR group were 85.66%, 72.78% and 61.45%, respectively. For patients in the SD group, the 1-year, 3-year, and 5-year DFS rates were 76.12%, 64.27% and 57.84%, respectively. Based on the TRG, 20 (9.05%) patients in the NAC group had TRG 0 grade. Significant differences in OS (P<0.05, **Figure 2G**) and DFS were observed between the TRG=0 and TRG ≠ 0 groups (P<0.05, **Figure 2H**). The 1-year, 3-year, and 5-year OS and DFS rates for patients in the TRG=0 group were all 95.00%. For patients in the TRG ≠ 0 group, the 1-year, 3-year, and 5-year OS rates were 93.45%, 77.02% and 68.57%, respectively. The 1-year, 3-year, and 5-year DFS rates were 81.27%, 66.98% and 53.03%, respectively.

### Analysis of Perioperative Outcomes

In the NAC and S groups, 208 (94.12%) and 197 (89.14%) patients underwent R0 resection, respectively. In addition, 10 (4.52%) patients received R1 resection, and 3 (1.36%) patients received R2 resection in the NAC group. In the S group, 13 (5.88%) and 11 (4.98%) patients underwent R1 and R2 resection, respectively. The median of dissected lymph nodes numbers in the NAC and S groups were 34 and 38, respectively. There were no significant differences in the radical degrees of operation and numbers of dissected lymph nodes between the two groups (P=0.07 and P=0.124, **Table 4**).

**Table 4:** Comparison of perioperative outcomes between NAC and S Groups after 1:1 matched.

Characteristics	Total (N = 442)	CSC (n = 221)	SC (n = 221)	P
**Radical degrees (n[%])**				0.072*
R0	405 (91.63)	208 (94.12)	197 (89.14)	
R1	23 (5.20)	10 (4.52)	13 (5.88)	
R2	14 (3.17)	3 (1.36)	11 (4.98)	
**No. of dissected lymph nodes**				0.124^§^
Median (range)	36 (0-121)	34 (0-104)	38 (9-121)	
**Postoperative hospital stays (d)**				<0.001^§^
Median (range)	12 (7-75)	11 (7-68)	13 (7-75)	
**Postoperative complications (n[%])**				0.037*
Yes	43 (9.73)	15 (6.79)	28 (12.67)	
No	399 (90.27)	206 (93.21)	193 (87.33)	

*χ^2^ test (compares the counts of categorical responses between 2 or more independent groups).

^§^Mann-Whitney rank test (a nonparametric alternative to the 2 sample t test compares the means of 2 independent groups).

Considering the postoperative hospital stays and postoperative complications, there were significant differences between the NAC and S groups (P<0.05, **Table 4**). The median postoperative hospital stays were 11 and 13 days in the NAC and S groups, respectively. The shortest postoperative hospital stay for both groups was 7 days. The longest postoperative hospital stays for the NAC and S groups were 68 and 75 days, respectively. The patient with a postoperative hospital stays of 68 days experienced intraperitoneal hemorrhage and underwent a second operation for hemostasis. The patient with a postoperative hospital stays of 75 days experienced anastomotic leakage, which was improved by conservative treatment.

From the perspective of postoperative complications, 15 (6.79%) patients in the NAC group experienced complications after the operation. Two patients underwent a second surgery due to the complications of anastomotic leakage and intraperitoneal hemorrhage. In the S group, 28 (12.67%) patients experienced postoperative complications. Four patients underwent a second surgery to treat complications, including anastomotic leakage, intraperitoneal hemorrhage, intestinal obstruction and pancreatic fistula. Details of the postoperative complications are given in **Table 5**.

**Table 5:** Details of the postoperative complications.

Postoperative Complications	NAC (n=15)	S (n=28)
**Incision infection**	3	3
**Anastomotic leakage**	3	9
**Duodenal stump fistula**	1	1
**Pancreatic fistula**	1	1
**Lymphatic fistula**	1	0
**Intra-abdominal infection**	2	1
**Intraperitoneal hemorrhage**	1	3
**Gastroparesis**	1	0
**Intestinal obstruction**	0	3
**Anastomotic stenosis**	1	0
**Pleural effusion**	1	1
**Pulmonary infection**	0	4
**Deep venous thrombosis**	0	2

## Discussion

Currently, the standard treatment for LAGC (cT4NxM0) is a combination of D2 gastrectomy and PCT. The chemotherapy regimens have changed over time. In the past decade, based on the results of MAGIC (15) and REAL-2 (30) studies, EOX had been the main NAC regimen in this study. In recent years, the German scholars advocated FLOT regimen (17, 31). Nowadays, FLOT has been recommended for NAC at a higher level than ECF and its modifications (8). Besides, SOX and XELOX are recommended at the same level as FLOT (Evidence 2A) (8). However, as a part of PCT, the value of NAC in improving OS and DFS is unclear. Therefore, we carried out this study to investigate this topic in China. In our study, a total of 902 patients were eligible for participation. The patients were divided into the following two groups according to whether they received NAC: the NAC (n=285) and S (n=617) groups. All patients underwent D2 gastrectomy and AC. The statistical analysis showed that there were several significant differences in the baseline characteristics between the two groups (**Table 1**). Because of these significant differences at baseline, we conducted propensity score matching (1:1 ratio) to minimize the differences in underlying confounding factors between the two groups.

After propensity score matching, we obtained 221 matched pairs of patients and there were no significant differences between the NAC and S groups (P>0.05, **Table 2**). In the Kaplan-Meier analysis, the survival curve showed that the OS and DFS rates of patients in the NAC group were significantly higher than those in the S group (P<0.0001, **Figures 2C, D**). Compared to those patients in the S group, the 1-year, 3-year, and 5-year OS rates for patients in the NAC group were increased by 9.88%, 32.92% and 36.07%, respectively. The 1-year, 3-year, and 5-year DFS rates for patients in the NAC group were also increased by 12.09%, 29.88% and 27.66%, respectively. We consider that the difference in survival between the two groups is due to whether or not NAC was used. NAC can promote tumor downstaging, eliminate potential micrometastasis, and improve patients’ prognosis. In our study, the 3-year DFS rate for patients in the NAC group was similar to the results in the PRODIGY (18) and RESOLVE (19) studies (69.74% *vs* 66.3% *vs* 62.02%). However, the 5-year OS rate for patients in the NAC group was significantly higher than that in the MAGIC (15) and FNCLCC & FFCD (16) trials (72.29% *vs* 36.6% *vs* 38%). The main reason for the difference in OS rate between studies may be the radical degrees of the operation. In our study, 94.12% of patients in the NAC group underwent R0 resection. In the MAGIC and FNCLCC & FFCD trials, only 69.3% and 84% of patients in the NAC groups obtained R0 resection, respectively.

**Figure 3:**
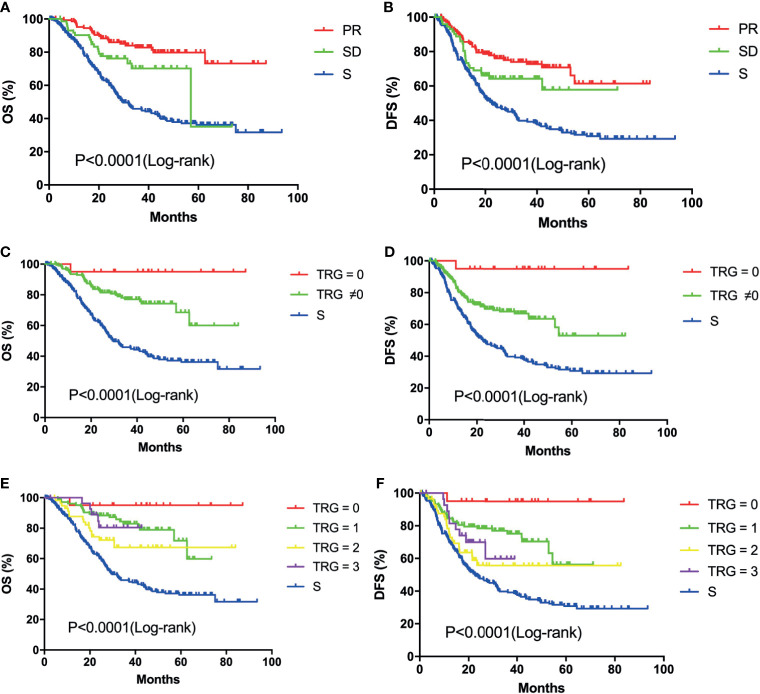
Kaplan-Meier survival curves of overall survival and disease-free survival: OS **(A)** and DFS **(B)** analysis of patients in the PR (n=148), SD (n=73) and S (n=221) groups; OS **(C)** and DFS **(D)** analysis of patients in the TRG = 0 (n=20), TRG ≠0 (n=201) and S (n=221) groups; OS **(E)** and DFS **(F)** analysis of patients in the different TRG grade (0, n=20; 1, n=107; 2, n=62; 3, n=32) and S (n=221) groups;.

In addition, subgroup analysis in the NAC group was conducted for further investigation. According to the RECIST, no one in the NAC group received CR in this study. Because it is difficult to distinguish a residual tumor from necrosis or fibrosis on imaging. Several patients received PD after NAC, but most of them did not receive surgery. One of the inclusion criteria in this study was that all patients received D2 gastrectomy. Therefore, only one PD patient was enrolled in this study, however this patient did not get matched during propensity score matching. Hence, survival was compared between PR and SD groups. The OS rate in the PR group was significantly higher than that in the SD group (P<0.05, **Figure 2E**) and no significant difference was found in the DFS rate between the two groups (P=0.07, **Figure 2F**). Compared with those for patients in the SD group, the 1-year, 3-year, and 5-year OS rates for patients in the PR group were increased by 4.93%, 12.62% and 44.73%, respectively. The OS and DFS rates in the PR and SD groups were all significantly higher than those in the S group (P<0.0001, **Figures 3A, B**). This result showed that patients with LAGC who achieved a disease response or stable after NAC treatment could benefit from NAC.

In addition, survival analysis was conducted to compare the survival of patients in different TRG groups. Previous studies had shown that LAGC patients with a well TRG would have better survival than those with no response or minor pathologic changes (32, 33). TRG is considered as an important predictor of survival in LAGC. However, there are a lot of factors influencing patients’ prognosis, such as the radical degree of surgery, adjuvant chemotherapy, postoperative complications and postoperative nutritional status. In this study, the OS and DFS rates of patients in the TRG=0 group were significantly higher than those in the TRG≠0 group (P<0.05, **Figures 2G, H**). For patients in the TRG=0 group, the 1-year, 3-year, and 5-year OS rates were improved by 1.55%, 17.98% and 26.43%, respectively, compared with those in the TRG≠0 group. The 1-year, 3-year, and 5-year DFS rates in the TRG=0 group were also increased by 13.73%, 28.02% and 41.97%, respectively. The OS and DFS rates in the different TRG grades were all significantly higher than those in the S group (P<0.0001, **Figures 3C–F**). This result suggested that better tumor regression in LAGC was associated with longer survival and lower rates of local recurrence.

When comparing perioperative outcomes between the NAC and S groups, the study showed that there was no significant difference in radical degrees of operation. However, patients in the NAC group had shorter postoperative hospital stays and lower postoperative complications than patients in the S group. This may be associated with improved nutritional status and reduced tumor burden after NAC, which are beneficial to postoperative recovery.

In conclusion, the results of our study showed that NAC can further improve prognosis and prevent recurrence in LAGC (cT4NxM0) patients. NAC is feasible and safe for LAGC (cT4NxM0) patients and does not increase the risk of perioperative surgery. Because our study is a retrospective study, it has certain limitations. A larger sample size of prospective, randomized, controlled clinical trial is necessary for the validation of this result.

## Data Availability Statement

The raw data supporting the conclusions of this article will be made available by the authors, without undue reservation.

## Ethics Statement

The studies involving human participants were reviewed and approved by the Ethics Committee of Ruijin Hospital affiliated to Shanghai Jiao Tong University School of Medicine. The patients/participants provided their written informed consent to participate in this study.

## Author Contributions

WX conceived and designed the study, analyzed the data and wrote the paper. LW and CY participated in the design of the study and edited the final paper. CH, SL, ZN, ZH, ZLZ, BS, ZY, and YZ collected the clinicopathological factors of all patients. RF, CL, XY, and MC followed up the patient’s survival status. WL revised the paper. MY and ZGZ gave professional guidance. All authors contributed to the article and approved the submitted version.

## Funding

This work was supported by National Natural Science Foundation of China (NO. 81772518) and Multicenter Clinical Trial of Shanghai Jiao Tong University School of Medicine (DLY201602 and 2018CR003).

## Conflict of Interest

The authors declare that the research was conducted in the absence of any commercial or financial relationships that could be construed as a potential conflict of interest.

## Publisher’s Note

All claims expressed in this article are solely those of the authors and do not necessarily represent those of their affiliated organizations, or those of the publisher, the editors and the reviewers. Any product that may be evaluated in this article, or claim that may be made by its manufacturer, is not guaranteed or endorsed by the publisher.
